# Complete Genome Sequence of Sin4, a Siphophage Infecting Carbapenemase-Producing Klebsiella pneumoniae

**DOI:** 10.1128/MRA.01048-19

**Published:** 2019-09-26

**Authors:** Micah Castillo, Rainie Tran, Heather Newkirk, Mei Liu, Jason J. Gill, Jolene Ramsey

**Affiliations:** a Center for Phage Technology, Texas A&M University, College Station, Texas, USA; Loyola University Chicago

## Abstract

Klebsiella pneumoniae is a commonly antibiotic-resistant human pathogen. This report describes the complete genome sequence and important features of Sin4, a siphophage infecting carbapenemase-producing K. pneumoniae. By its genome size, predicted packaging mechanism, protein similarity, and classification given to its closest relatives, Sin4 was determined to be a T1-like phage.

## ANNOUNCEMENT

Carbapenemase-producing K. pneumoniae is a pathogenic Gram-negative bacterium within the *Enterobacteriaceae* family of microorganisms (1). Sequence type 258 (ST258), a multidrug-resistant clade spreading in hospital settings around the world, was used here to isolate new bacteriophages from the environment (2). We report here the complete genome sequence of the K. pneumoniae siphophage Sin4.

Bacteriophage Sin4 was isolated from a filtered (0.2-μm pore size) sample collected at a wastewater treatment plant in College Station, TX, based on its ability to grow on a pKpQIL plasmid-cured derivative of K. pneumoniae strain 1776c (2). The K. pneumoniae host was grown aerobically in tryptic soy broth or agar (Difco) at 37°C, and phage propagation was done using the soft agar overlay method (3). Morphology was determined using transmission electron microscopy performed at the Texas A&M University Microscopy and Imaging Center on 2% (wt/vol) uranyl acetate-stained samples (4). Phage genomic DNA was prepared by the shotgun library preparation protocol modification of the Promega Wizard DNA clean-up system (5). Library preparation was done using a TruSeq Nano low-throughput kit, and sequencing occurred on an Illumina MiSeq platform with v2 500-cycle chemistry. There were 707,310 total paired-end 250-bp reads in the phage-containing index. The reads were quality controlled using FastQC (http://www.bioinformatics.babraham.ac.uk/projects/fastqc/). After trimming with the FASTX-Toolkit 0.0.14 (http://hannonlab.cshl.edu/fastx_toolkit/), a single contig was assembled at 983-fold coverage using SPAdes v3.5.0 (6). The genome was confirmed to be complete and accurate by Sanger sequencing of a PCR product off the contig ends (forward primer, 5′-CCGAAAGGCCTGGTATAGTT-3′, and reverse primer, 5′-CAGTCTGCTTGTCGTTGATTTG-3′). The program PhageTerm predicts that Sin4 uses a headful packaging mechanism (7). To identify Rho-independent terminators, the program TransTermHP v2.09 was used (8). Protein-coding genes were predicted using Glimmer v3.0 and MetaGeneAnnotator v1.0 and corrected using tools available on the Center for Phage Technology Galaxy instance with Web Apollo (https://cpt.tamu.edu/galaxy-pub) (9–12). According to an ARAGORN v2.36 scan, Sin4 does not contain tRNA genes (13). The prediction of protein function was performed using primarily InterProScan v5.22-61 and BLAST v2.2.31 with the NCBI nonredundant and UniProtKB Swiss-Prot/TrEMBL databases (14–16). Additionally, TMHMM v2.0 for transmembrane domains and HHpred (multiple sequence alignment [MSA] generation with the HHblits ummiclus30_2018_08 database and modeling with PDB_mmCIF70, HHSuite v3.0) predictions provided further support for the annotation (17, 18). Unless otherwise stated, default parameters were used for all tools listed.

Sin4 is a siphophage with a 49,916-bp genome, a coding density of 91.6%, and a G+C content of 50.3%. Of the 78 predicted genes in Sin4, 42 are not assigned a function. Sin4 is most closely related to Klebsiella phage 1513 (GenBank accession number KP658157), with which it shares 85.4% nucleotide sequence identity across the entire genome according to progressiveMauve v2.4.0 (19). Phages 1513 and Sin4 also share 69 proteins.

By genome size, predicted packaging mechanism, and the classification given to its closest relatives, Sin4 is a T1-like phage with 42 proteins similar to phage T1 (GenBank accession number NC_005833) (20). Notable Sin4 genes include those for a DNA cytosine methyltransferase (NCBI accession number QEG07100) and a DNA adenine methyltransferase (NCBI accession number QEG07085).

### Data availability.

The genome sequence and associated data for phage Sin4 were deposited under GenBank accession number MK931442, BioProject accession number PRJNA222858, SRA accession number SRR8869237, and BioSample accession number SAMN11360409.

## References

[1] Logan LK, Weinstein RA 2017 The epidemiology of carbapenem-resistant Enterobacteriaceae: the impact and evolution of a global menace. J Infect Dis 215:S28–S36. doi:10.1093/infdis/jiw282. 28375512 PMC5853342

[2] Satlin MJ, Chen L, Patel G, Gomez-Simmonds A, Weston G, Kim AC, Seo SK, Rosenthal ME, Sperber SJ, Jenkins SG, Hamula CL, Uhlemann A-C, Levi MH, Fries BC, Tang Y-W, Juretschko S, Rojtman AD, Hong T, Mathema B, Jacobs MR, Walsh TJ, Bonomo RA, Kreiswirth BN 2017 Multicenter clinical and molecular epidemiological analysis of bacteremia due to carbapenem-resistant Enterobacteriaceae (CRE) in the CRE epicenter of the United States. Antimicrob Agents Chemother 61:e02349-16. doi:10.1128/AAC.02349-16. 28167547 PMC5365653

[3] Adams MH 1956 Bacteriophages. Interscience Publishers, Inc, New York, NY.

[4] Valentine RC, Shapiro BM, Stadtman ER 1968 Regulation of glutamine synthetase. XII. Electron microscopy of the enzyme from Escherichia coli. Biochemistry 7:2143–2152. doi:10.1021/bi00846a017. 4873173

[5] Summer EJ 2009 Preparation of a phage DNA fragment library for whole genome shotgun sequencing. Methods Mol Biol 502:27–46. doi:10.1007/978-1-60327-565-1_4. 19082550

[6] Bankevich A, Nurk S, Antipov D, Gurevich AA, Dvorkin M, Kulikov AS, Lesin VM, Nikolenko SI, Pham S, Prjibelski AD, Pyshkin AV, Sirotkin AV, Vyahhi N, Tesler G, Alekseyev MA, Pevzner PA 2012 SPAdes: a new genome assembly algorithm and its applications to single-cell sequencing. J Comput Biol 19:455–477. doi:10.1089/cmb.2012.0021. 22506599 PMC3342519

[7] Garneau JR, Depardieu F, Fortier L-C, Bikard D, Monot M 2017 PhageTerm: a tool for fast and accurate determination of phage termini and packaging mechanism using next-generation sequencing data. Sci Rep 7:8292. doi:10.1038/s41598-017-07910-5. 28811656 PMC5557969

[8] Kingsford CL, Ayanbule K, Salzberg SL 2007 Rapid, accurate, computational discovery of Rho-independent transcription terminators illuminates their relationship to DNA uptake. Genome Biol 8:R22. doi:10.1186/gb-2007-8-2-r22. 17313685 PMC1852404

[9] Delcher AL, Harmon D, Kasif S, White O, Salzberg SL 1999 Improved microbial gene identification with GLIMMER. Nucleic Acids Res 27:4636–4641. doi:10.1093/nar/27.23.4636. 10556321 PMC148753

[10] Noguchi H, Taniguchi T, Itoh T 2008 MetaGeneAnnotator: detecting species-specific patterns of ribosomal binding site for precise gene prediction in anonymous prokaryotic and phage genomes. DNA Res 15:387–396. doi:10.1093/dnares/dsn027. 18940874 PMC2608843

[11] Afgan E, Baker D, Batut B, van den Beek M, Bouvier D, Cech M, Chilton J, Clements D, Coraor N, Grüning BA, Guerler A, Hillman-Jackson J, Hiltemann S, Jalili V, Rasche H, Soranzo N, Goecks J, Taylor J, Nekrutenko A, Blankenberg D 2018 The Galaxy platform for accessible, reproducible and collaborative biomedical analyses: 2018 update. Nucleic Acids Res 46:W537–W544. doi:10.1093/nar/gky379. 29790989 PMC6030816

[12] Lee E, Helt GA, Reese JT, Munoz-Torres MC, Childers CP, Buels RM, Stein L, Holmes IH, Elsik CG, Lewis SE 2013 Web Apollo: a Web-based genomic annotation editing platform. Genome Biol 14:R93. doi:10.1186/gb-2013-14-8-r93. 24000942 PMC4053811

[13] Laslett D, Canback B 2004 ARAGORN, a program to detect tRNA genes and tmRNA genes in nucleotide sequences. Nucleic Acids Res 32:11–16. doi:10.1093/nar/gkh152. 14704338 PMC373265

[14] Jones P, Binns D, Chang H-Y, Fraser M, Li W, McAnulla C, McWilliam H, Maslen J, Mitchell A, Nuka G, Pesseat S, Quinn AF, Sangrador-Vegas A, Scheremetjew M, Yong S-Y, Lopez R, Hunter S 2014 InterProScan 5: genome-scale protein function classification. Bioinformatics 30:1236–1240. doi:10.1093/bioinformatics/btu031. 24451626 PMC3998142

[15] Camacho C, Coulouris G, Avagyan V, Ma N, Papadopoulos J, Bealer K, Madden TL 2009 BLAST+: architecture and applications. BMC Bioinformatics 10:421. doi:10.1186/1471-2105-10-421. 20003500 PMC2803857

[16] The UniProt Consortium. 2018 UniProt: the universal protein knowledgebase. Nucleic Acids Res 46:2699. doi:10.1093/nar/gky092. 29425356 PMC5861450

[17] Krogh A, Larsson B, Heijne von G, Sonnhammer EL 2001 Predicting transmembrane protein topology with a hidden Markov model: application to complete genomes. J Mol Biol 305:567–580. doi:10.1006/jmbi.2000.4315. 11152613

[18] Zimmermann L, Stephens A, Nam S-Z, Rau D, Kübler J, Lozajic M, Gabler F, Söding J, Lupas AN, Alva V 2018 A completely reimplemented MPI Bioinformatics Toolkit with a new HHpred server at its core. J Mol Biol 430:2237–2243. doi:10.1016/j.jmb.2017.12.007. 29258817

[19] Darling AE, Mau B, Perna NT 2010 ProgressiveMauve: multiple genome alignment with gene gain, loss and rearrangement. PLoS One 5:e11147. doi:10.1371/journal.pone.0011147. 20593022 PMC2892488

[20] Roberts MD, Martin NL, Kropinski AM 2004 The genome and proteome of coliphage T1. Virology 318:245–266. doi:10.1016/j.virol.2003.09.020. 14972552

